# Comparison of clinicopathologic parameters and oncologic outcomes between type 1 and type 2 papillary renal cell carcinoma

**DOI:** 10.1186/s12894-020-00716-0

**Published:** 2020-09-15

**Authors:** Xiang Le, Xiang-Bo Wang, Hao Zhao, Ren-Fu Chen, Peng Ge

**Affiliations:** 1 Department of Urology, the Affiliated Hospital of Xuzhou Medical University, Xuzhou, Jiangsu China; 2 Department of Pathology, the Affiliated Hospital of Xuzhou Medical University, Xuzhou, Jiangsu China

**Keywords:** Papillary renal cell carcinoma, WHO/ISUP grade, Prognosis, Subtype

## Abstract

**Background:**

To compare the clinicopathologic parameters and oncologic outcomes between type 1 and type 2 papillary renal cell carcinoma (PRCC).

**Methods:**

This study was approved by the review board (NO.XYFY2019-KL032–01). Between 2007 and 2018, 52 consecutive patients who underwent surgery at a single tertiary referral hospital were included. Clinicopathologic and survival data were collected and entered into a database. The Kaplan-Meier method, and univariate and multivariate Cox proportional hazard regression analyses were performed to estimate progression-free survival (PFS) and cancer-specific survival (CSS).

**Results:**

Of the 52 patients, 24 (46.2%) were diagnosed with type 1 PRCC, and 28 (53.8%) had type 2 PRCC. The mean tumor size was 4.8 ± 2.5 cm. The two subtypes displayed different morphological features: foamy macrophages were more common in type 1 PRCC, while eosinophils and microvascular angiolymphatic invasion were more frequent in type 2 PRCC. Type 2 cases showed higher tumor stage and World Health Organization/International Society of Urological Pathology (WHO/ISUP) grade than type 1 cases (T3-T4: 43% vs 17%, *P* = 0.041; G3-G4: 43% vs 8%, *P* = 0.005). In univariate analysis, type 2 PRCC had a lower probability for PFS and CSS than patients with type 1 PRCC (*P* = 0.016, *P* = 0.049, log-rank test, respectively). In multivariate analysis, only WHO/ISUP grade (HR 11.289, 95% CI 2.303–55.329, *P* = 0.003) and tumor size (HR 1.244, 95% CI 1.034–1.496, *P* = 0.021) were significantly associated with PFS.

**Conclusions:**

PRCC subtype displayed different morphological features: foamy macrophages, eosinophils and microvascular angiolymphatic invasion are pathologic features that may aid in the distinction of the two subtypes. Histologic subtype of PRCC is not an independent prognostic factor and only WHO/ISUP grade and tumor size were independent predictors for PFS.

## Background

Papillary renal cell carcinoma (PRCC), the second most common renal cell carcinoma (RCC) following clear cell RCC, accounts for 6 to 18% of all RCC cases [1]. It is a markedly heterogeneous entity characterized by different histologic subtypes, disease progression and clinical outcomes [2, 3]. Delahunt and Eble initially subclassified the PRCC into type 1 and type 2 based on morphological and immunohistochemical features in 1997 [4]. Typically, type 1 demonstrates the papillae covered by a single layer of simple cuboidal epithelia with scant cytoplasm, while type 2 is characterized by the presence of nuclear pseudostratifcation [5].

Recently, Magers et al. showed that type 1 PRCC was more likely to have clear cytoplasm and nuclear grooves, whereas type 2 PRCC had more abundant, granular cytoplasm and a higher nuclear grade [6]. Leroy et al. suggested that MUC1 immunostaining was usually positive in type 1 PRCC, which might be helpful in classifying such tumors [7]. However, due to the overlaps in morphologic, immunohistochemical and molecular features, it is difficult to classify all PRCCs into subtypes absolutely with current controversial criteria [2, 6, 8].

The issue whether the histologic subtypes affect oncological outcomes remains in debate. Some studies suggested type 1 PRCC was associated with a lower nuclear grade, lower tumor stage, and better prognosis than type 2 PRCC [9]. However, some other studies showed almost similar prognosis across the two subtypes [10, 11].

Herein, we performed the present study in an attempt to compare the clinicopathologic parameters and oncologic outcomes between type 1 and type 2 PRCC using a homogeneous pathological entity.

## Methods

### Patient population and pathologic evaluation

This was a retrospective, single-institution study approved by the Affiliated Hospital of Xuzhou Medical University review board (NO.XYFY2019-KL032–01). A total of 56 tumors initially diagnosed as PRCC between 2007 and 2018 at a single tertiary hospital were evaluated. All hematoxylin and eosin–stained slides available were reviewed by the same pathologist (author Xiang-Bo Wang). Histologic subtypes were recorded according to the WHO classification of kidney tumors [12]. Four cases were excluded from the study after review. The remaining 52 patients were included in the present study.

Because of the difficulties in validation, reproducibility, and interpretation of the Fuhrman system [13], all samples were re-graded according to the four-tiered World Health Organization/International Society of Urological Pathology (WHO/ISUP) grading system [14, 15]. Pathological stage was assigned according to the American Joint of Committee on Cancer staging manual [10]. More morphological parameters were also recorded, including foamy macrophages, hemosiderin laden macrophages, necrosis, sarcomatoid differentiation, eosinophils, hyaline cells, classic papillary architecture, solid architecture, tubular architecture, perinephric/renal sinus fat invasion, and microvascular angiolymphatic invasion [6, 16].

Patient’s clinicopathologic characteristics were entered into a database. Postoperative follow-up was not standardized. Generally, patients were evaluated every 3 months during the first year, every 6 months during the next 2 years, and then annually. Follow-up included physical examinations, laboratory measurements and imaging studies unless otherwise clinically indicated. The last follow-up was performed in May 2019. The outcomes of interest were progression-free survival (PFS) and cancer-specific survival (CSS). PFS was defined as the time from the date of surgery to local recurrence or distant metastasis and CSS was defined as the time from the date of surgery to a kidney cancer-related death. Surviving patients were censored at the last follow-up.

### Statistical analysis

The continuous variables were compared using the independent-sample t test and the categorical variables were compared using the χ^2^ - test. The Kaplan-Meier method was used to estimate the impacts of the subclassification and clinicopathologic parameters of PRCC on PFS and CSS, and the comparison was made with log-rank test. Univariate and multivariate (stepwise selection with enter and remove limits of *P* = 0.05 and *P* = 0.05, respectively) Cox proportional hazards regression analyses were performed to evaluate independency of prognostic factors for PFS and CSS. Nine variables (gender, age, WHO/ISUP grade, cell-based immunotherapy, type of surgery, surgical approach, pathological stage, PRCC subtypes and tumor size) were used in the stepwise selection analysis. Two-sided *P* < 0.050 was considered to indicate a statistically significant difference. Statistical analysis was performed using the Statistical Analysis System version 9.4 (SAS Institute, Cary, NC, USA) or Statistical Package for the Social Sciences 25.0 software (SPSS Inc., Chicago, IL, USA).

## Results

### Association with clinicopathologic characteristics

**Fig. 1:**
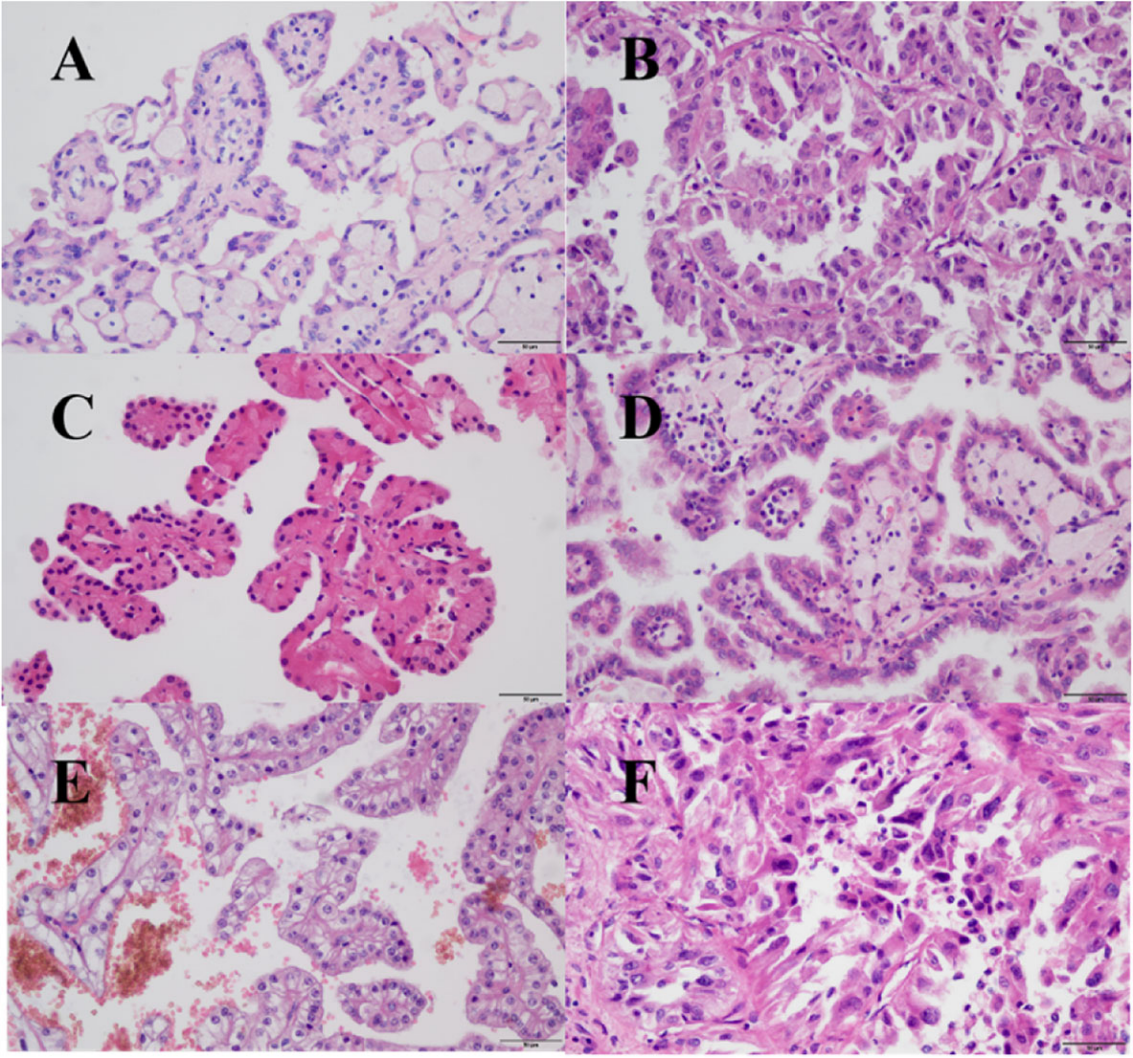
Papillary renal cell carcinoma grading and types. **a**, type 1; **b**, type 2; Papillary renal cell carcinoma graded as nucleolar grade 1(**c**), 2(**d**), 3(**e**), 4(**f**). Hematoxylin and eosin stains, original magnification × 400

After histopathological review, the diagnosis of PRCC was confirmed in a total of 52 patients, including 38 males and 14 females (Table 1). The mean tumor size was 4.8 ± 2.5 cm. There were 24 (46.2%) patients with type 1 PRCC and 28 (53.8%) patients with type 2 PRCC. The age at surgery ranged from 17 to 86 years old (mean: 55.1). According to Table 1, patients with type 2 PRCC were more likely to have a higher WHO/ISUP grade (G3–4 in type 2 vs type 1: 43% vs 8%, *P* = 0.005) and an advanced tumor stage (T3–4 in type 2 vs type 1: 43% vs 17%, *P* = 0.041, Fig. 1). Moreover, patients with type 2 PRCC were more likely to receive radical nephrectomy (61% vs 33%; *P* = 0.049). Other clinical characteristics (including gender distribution, age, tumor size, and cell-based immunotherapy) demonstrated no statistically significant differences across the two groups.

**Table 1:** Clinical characteristics between type 1 and type 2 papillary renal cell carcinoma

	Type 1 PRCC	Type 2 PRCC	P
Gender			
Male	18 (75.0%)	20 (71.4%)	0.772
Female	6 (25.0%)	8 (28.6%)	
Age (years)	53.4 ± 12.6	55.8 ± 15.5	0.552
Tumor size (cm)	4.6 ± 2.26	4.9 ± 2.76	0.683
Cell-based immunotherapy^a^			
Yes	3 (12.5%)	5 (17.9%)	0.882
No	21 (87.5%)	23 (82.1%)	
WHO/ISUP grade			
G1/G2	22 (91.7%)	16 (57.1%)	0.005
G3/G4	2 (8.3%)	12 (42.9%)	
Tumor stage (T)			
T1/T2	20 (83.3%)	16 (57.1%)	0.041
T3/T4	4 (16.7%)	12 (42.9%)	
Type of surgery			
Radical nephrectomy	8 (33.3%)	17 (60.7%)	0.049
Partial nephrectomy	16 (66.7%)	11 (39.3%)	
Surgical approach			
Open surgery	3 (12.5%)	9 (32.1%)	0.094
Laparoscopic surgery	21 (87.5%)	19 (67.9%)	
Disease progression			
Yes	1 (4.0%)	9 (33.3%)	0.028
No	22 (96.0%)	18 (66.7%)	
Death from tumor			
Yes	0 (0.0%)	6 (22.2%)	0.048
No	23 (100.0%)	21 (77.8%)	

*Abbreviations*: *WHO/ISUP* World Health Organization/International Society of Urological Pathology, *PRCC* Papillary renal cell carcinoma

^a^Cell-based immunotherapy refers to cytokine-induced killer cells therapy

Table 2 displays the pathologic characteristics of the patients. Presence of foamy macrophages was more frequent in type 1 (83%) than in type 2 (57%) (*P* = 0.041). In contrast, eosinophils and microvascular angiolymphatic invasion were more frequent in type 2 PRCC (*P* = 0.012 and *P* = 0.028, respectively). There were no statistically significant differences across the two groups in terms of hemosiderin laden macrophages, necrosis, sarcomatoid differentiation, hyaline cells, classic papillary architecture, solid architecture, tubular architecture, and perinephric/renal sinus fat invasion.

**Table 2:** Pathologic characteristics between type 1 and type 2 papillary renal cell carcinoma

	Type 1	Type 2	P
Foamy macrophages			
Yes	20 (83.3%)	16 (57.1%)	0.041
No	4 (16.7%)	12 (42.9%)	
Hemosiderin laden macrophages			
Yes	20 (83.3%)	27 (96.4%)	0.261
No	4 (16.7%)	1 (3.6%)	
Necrosis			
Yes	11 (45.8%)	18 (64.3%)	0.182
No	13 (54.2%)	10 (35.7%)	
Sarcomatoid differentiation			
Yes	0 (0.0%)	2 (7.1%)	0.493
No	24 (100.0%)	26 (92.9%)	
Eosinophils			
Yes	7 (29.2%)	18 (64.3%)	0.012
No	17 (70.8%)	10 (35.7%)	
Hyaline cells			
Yes	12 (50.0%)	18 (64.3%)	0.299
No	12 (50.0%)	10 (35.7%)	
Classic papillary architecture			
Yes	23 (95.8%)	28 (100.0%)	0.462
No	1 (4.2%)	0 (0.0%)	
Solid architecture			
Yes	4 (16.7%)	10 (35.7%)	0.123
No	20 (83.3%)	18 (64.3%)	
Tubular architecture			
Yes	15 (62.5%)	16 (57.1%)	0.695
No	9 (37.5%)	12 (42.9%)	
Perinephric/renal sinus fat invasion			
Yes	3 (12.5%)	8 (28.6%)	0.157
No	21 (87.5%)	20 (71.4%)	
Microvascular angiolymphatic invasion			
Yes	1 (4.2%)	9 (32.1%)	0.028
No	23 (95.8%)	19 (67.9%)	

### Association with survival

Two patients were lost to follow-up after surgery. Thus, the remaining 50 patients were included in survival analysis. The mean follow-up duration was 38 months (standard error, 4.7). Overall, 9 (33%) and 1 (4%) patients with type 2 PRCC and type 1 PRCC underwent disease progression, respectively (*P* = 0.028, Table 1). A total of 6 patients died of PRCC. The most common recurrence or metastatic sites were retroperitoneal /distant lymph node, lung, liver, and retroperitoneal cavity (Table 3).

**Table 3:** Recurrence and/or metastatic site in disease progression patients

Patient ID	Recurrence and/or metastatic site
1	bone
2	distant lymph node
3	liver
4	retroperitoneal lymph node, bone
5	retroperitoneal lymph node, liver, bone
6	retroperitoneal lymph node, rectum, retroperitoneal cavity
7	lung,bone, brain
8	retroperitoneal lymph node, lung, spleen, retroperitoneal cavity, abdominopelvic cavity, abdominal wall
9	lung
10	retroperitoneal cavity, adrenal gland, liver

**Fig. 2:**
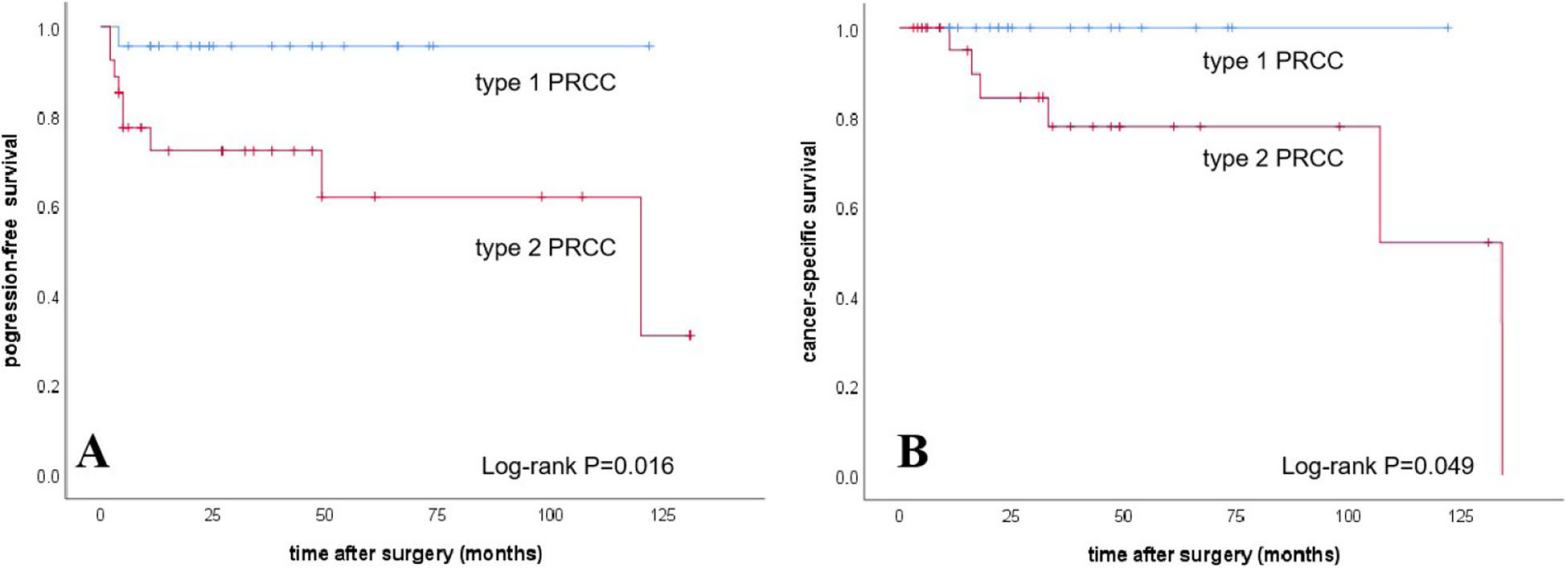
Kaplan-meier curves. **a**, progression-free survival. **b**, cancer-specific survival. *P*-values are based on log-rank test. PRCC, papillary renal cell carcinoma

Figure 2a exhibits the Kaplan–Meier plots for PFS estimates stratified by type 1 PRCC versus type 2. Type 2 PRCC patients had a lower probability for PFS than patients with type 1 (*P* = 0.016, log-rank test, Fig. 2a; *P* = 0.045, univariate Cox analysis, Table 4). Similarly, patients with type 2 PRCC were at significantly higher risk for cancer specific mortality than patients with type 1 PRCC (*P* = 0.049, Fig. 2b).

**Table 4:** Univariate Cox analysis on progression-free survival in papillary renal cell carcinoma patients

	HR	95%CI	P
Gender			
Female	1		
Male	0.364	0.095–1.389	0.139
Age			
≤ 60	1		
>60	0.885	0.248–3.156	0.851
Surgical approach			
Open surgery	1		
Laparoscopic surgery	0.323	0.086–1.205	0.092
Type of surgery			
Partial nephrectomy	1		
Radical nephrectomy	0.092	0.012–0.726	0.024
Cell-based immunotherapy			
No	1		
Yes	1.132	0.236–5.425	0.877
Tumor stage			
T1/T2	1		
T3/T4	12.136	2.557–57.599	0.002
WHO/ISUP grade			
G1/G2	1		
G3/G4	13.692	2.890–64.874	0.001
PRCC subtype			
type 1	1		
type 2	8.326	1.050–66.024	0.045
Tumor size	1.340	1.117–1.608	0.002

*Abbreviations*: *HR* Hazard ratio, *CI* Confidence interval, *WHO/ISUP* World Health Organization/International Society of Urological Pathology, *PRCC* Papillary renal cell carcinoma

Multivariate analysis was performed using a Cox regression with stepwise selection of covariates. Among the nine variables (gender, age, WHO/ISUP grade, cell-based immunotherapy, type of surgery, surgical approach, pathological stage, PRCC subtypes and tumor size), only WHO/ISUP grade (HR 11.289, 95% CI 2.303–55.329, *P* = 0.003) and tumor size (HR 1.244, 95% CI 1.034–1.496, *P* = 0.021) entered the model. The histologic subtype of PRCC was not an independent predictor for PFS. In view of the fact that the statistical power was weakened by very few death events, multivariate Cox proportional hazards regression analyses were not performed for CSS.

## Discussion

PRCC represents the largest subset of non-clear cell RCC [17]. It is traditionally subdivided into 2 subtypes on the basis of histomorphologic features. More recently, new PRCC subtype has been recognized [6]. In this current series, several morphological aspects of PRCC architecture and cytological features were investigated. We found that aggregates of foamy macrophages in the background stroma were common (69.2% of cases) and presence of foamy macrophages was less frequent in type 2 PRCC (57.1%) than type 1 PRCC (83.3%). Similar to our findings, Polifka et al. demonstrated that 70.9% of type 1 PRCC and 49.0% type 2 PRCC presented foamy macrophages and presence of foamy macrophages was linked with a better overall survival [18]. Unlike foamy macrophages, eosinophils and microvascular angiolymphatic invasion were more common in type 2 PRCC. Taken together, these findings in the present study may assist to discriminate between type 1 PRCC and type 2 PRCC.

The issue whether type 2 PRCC predicts poorer prognosis than type 1 PRCC remains controversial. Some claimed that type 2 has a more aggressive behavior than type 1, whereas others reported similar clinical course [9, 17–19]. From our perspectives, several points should be noted when we take the prior findings into consideration. Firstly, some studies investigated the prognosis of the subtypes after the data were adjusted against the effects of Fuhrman grade [10, 20]. Delahunt et al. indicated that Fuhrman grading was inappropriate for PRCC and recommended WHO/ISUP grading system [21]. Secondly, some investigations were based on multicenter database, thus without a centralized pathologic review, differing criteria and nomenclature likely resulted in under- or over-reporting of PRCC [10].

The present study was designed to investigate the outcomes of PRCC using a homogeneous pathological entity by minimizing potential confounding effects associated with different diagnosis criteria. We found that type 2 PRCC showed a higher tumor stage and a higher WHO/ISUP grade than type 1 PRCC. It indicates that type 2 PRCC tends to have a more aggressive course than type 1 PRCC, which is in accordance with the results of previous studies [9]. Regarding the prognostic factors, we found differences in terms of PFS and CSS between type 1 and type 2 in univariate analysis. However, multivariate analysis failed to draw a conclusion that histologic subtype was an independent predictor of PFS. Several explanations are possible for these somewhat contradictory results. Firstly, type 2 PRCC tends to run parallel with high WHO/ISUP grade and high tumor stage and is less strongly related to outcomes than pathologic features. Besides, our study was limited by an overall short follow-up time and a small sample size. The statistical power was weakened by very few events.

Recently, a novel grading system for clear cell RCC and PRCC was proposed by ISUP and then endorsed by the WHO [14, 21]. The applicability of WHO/ISUP grading system to PRCC has been validated and recommended widely [14, 18]. Cornejo et al. reported that WHO/ISUP grade was statistically superior to Fuhrman grade in predicting survival in both univariate and multivariate analyses [16]. In this study, all samples were re-graded according to the four-tiered WHO/ISPU grading system. In accordance with previous studies, we found that WHO/ISUP grade was an independent prognostic factor for PFS.

It is generally recognized that clear cell RCC conveys superior outcome than PRCC. However, different PRCC risk subgroups may not predict the same prognosis. Steffens and colleagues [3] indicated that PRCC could apparently be differentiated into organ-confined/localised and advanced/metastatic subgroup. Compared to clear cell RCC, the former subgroup had a significantly better prognosis, while the latter subgroup had a worse prognosis. Previous studies have shown that PRCC, especially type 2 PRCC, is an heterogeneous entity with divergent spectra [18]. Hence, our understanding of PRCC should not be limited to histologic subtypes. More information about genome profiles and cell signaling of tumor induction, promotion, and progression is required.

Although our study offers new information in answering the question of prognostic differences between patients with type 1 PRCC and patients with type 2 PRCC, it is not devoid of potential limitations that need to be acknowledged. First and foremost are the limitations inherent to its retrospective nature. In addition, in order to make a homogeneous pathological entity, we only chose those patients between 2007 and 2018, thus the follow-up time and sample size were the limitations of the present study.

## Conclusions

This study compared clinicopathologic parameters and oncologic outcomes between type 1 PRCC and type 2 PRCC in a homogeneous pathological entity. We found that foamy macrophages were more common in type 1 PRCC, while eosinophils and microvascular angiolymphatic invasion were more frequent in type 2 PRCC. Although type 2 PRCC tended to have a higher tumor stage and WHO/ISUP grade, histologic subtype was not an independent prognostic factor. In multivariate analysis, only WHO/ISUP grade and tumor size were significantly associated with PFS.

## Data Availability

The data supporting the conclusions used and/or analyzed in this study are available from the corresponding author by request.

## Abbreviations

PRCC: Papillary renal cell carcinoma
RCC: Renal cell carcinoma
WHO/ISUP: World Health Organization/International Society of Urological Pathology
PFS: Progression-free survival
CSS: Cancer-specific survival
